# Evaluation of Fecal Sample Pooling for Real-Time RT-PCR Testing SARS-CoV-2 in Animals

**DOI:** 10.3390/v16111651

**Published:** 2024-10-23

**Authors:** Antonio Leonardi-Cattolica, Sandipty Kayastha, Megan Miller, Jake Guag, Andriy Tkachenko, James Lowe, Matthew Allender, Karen Terio, Leyi Wang

**Affiliations:** 1 Veterinary Diagnostic Laboratory, Department of Veterinary Clinical Medicine, University of Illinois College of Veterinary Medicine, Urbana, IL 61802, USA; 2 Department of Pathobiology, University of Illinois at Urbana-Champaign, 2001 South Lincoln Ave, Urbana, IL 61802, USA; 3 Center for Veterinary Medicine, U.S. Food and Drug Administration, Laurel, MD 20708, USA; 4 Department of Veterinary Clinical Medicine, University of Illinois at Urbana-Champaign, 2001 South Lincoln Ave, Urbana, IL 61802, USA; 5 Zoological Pathology Program, University of Illinois College of Veterinary Medicine, Urbana, IL 61802, USA

**Keywords:** SARS-CoV-2, sample pooling, feces, RT-PCR, animals

## Abstract

During the COVID-19 pandemic, veterinary diagnostic laboratories tested both human and animal samples and needed to ensure that they could accurately perform large numbers of diagnostic tests in a timely manner. Sample pooling, a methodology used effectively for over 80 years as a surveillance tool for screening large numbers of potentially infected individuals, was employed. Given its sensitivity, real-time polymerase chain reaction (PCR) is more suitable for employing this strategy, as compared to other less sensitive testing methods. In this study, we evaluated the capability of detecting SARS-CoV-2 in both 5-sample and 10-sample pools of feces using real-time reverse transcriptase polymerase chain reaction (rRT-PCR) as well as determined the level of sensitivity. A blinded method test (BMT) by an independent laboratory was conducted to assess the five-sample fecal pool. To complement detection capability, the stability of the genome within a PBS fecal suspension was measured under various time and temperature conditions across a 28-day period. Our results showed that the limit of detection for 5-sample and 10-sample fecal pools is 12.8 and 6.4 genome copies in a 25 µL PCR, respectively. The 5-sample and 10-sample pooling resulted in a cycle threshold (Ct) value loss of 2.35 and 3.45, as compared to Ct values of known positive individual samples, but consistent detection was still achieved in pools containing positive samples with an original Ct below 36 and 34, respectively. The simulation of clinical five-sample pooling showed that all positive samples could be detected regardless of the number (1–3) of positive samples in each pool. The BMT results demonstrated excellent sensitivity (100 copies/reaction) in five-sample pools for the detection of SARS-CoV-2 RNA even though a fecal matrix effect was observed. Finally, our results show that the SARS-CoV-2 genome remains stable over a wide range of time and temperature variations. Overall, our findings provide solid data to scale up SARS-CoV-2 testing capacity in veterinary diagnostic laboratories.

## 1. Introduction

In late 2019, a novel coronavirus, severe acute respiratory syndrome coronavirus 2 (SARS-CoV-2), emerged. The virus quickly spread throughout the world and, at the date of this writing, has resulted in greater than 773 million human cases and over 7 million case-related fatalities [1]. Because of its cell receptor ACE2, the virus has a wide host range and has been detected genomically or serologically in at least thirty-eight animal species [2,3]. These animal species identified as positive for SARS-CoV-2 are classified into four groups: domestic/pet, zoo (captive), wild, and farm animals [4]. Unsurprisingly, SARS-CoV-2 is also capable of zoonotic transmission, and spillback events were reported in different studies [5,6,7,8]. As a result, for humans, concern not only centers around situations in which susceptible people have contact with those who are infected, but also when susceptible humans and infected animals are in close proximity, e.g., companion animals/owners, production animals/workers, zoo animals/caretakers, etc.

To help characterize the extent of the COVID-19 pandemic, as well as enhance the public health response, the strategy of pooling samples was widely implemented. Sample pooling is a diagnostic strategy introduced in the 1940s for detecting syphilis in United States soldiers during World War II [9]. The principle is based on the Wassermann test and involves combining small portions of multiple samples, which altogether equal the amount required to perform the test, i.e., using the “pooled samples” to run a single test. This allows for the identification of a single infected individual, or a few infected individuals, from within a larger number of samples, without testing each sample individually. When a positive is detected, testing of the individual samples is then conducted to determine which individual(s) in the group are true positives. This application allows for rapidly testing mass individuals while using fewer resources and saving time. Sample pooling has been used in clinical testing for HIV, influenza, and Malaria [10,11,12]. In the veterinary field, sample pooling has been commonly used for testing pathogens such as bovine viral diarrhea virus (BVDV), avian influenza virus (AIV), Newcastle disease virus (NDV), and porcine reproductive and respiratory syndrome virus (PRRSV). Sample pooling has also been studied for testing *Mycoplasma hyoneumonia* in pigs, suggestive of a useful approach for a herd eradication program [13]. During the COVID-19 pandemic, the pooling of nasopharyngeal samples was adopted for human SARS-CoV-2 screening and proved to be both cost-effective and efficient [14]. Studies on SARS-CoV-2 have demonstrated that the diagnostic performance of a sample pooling-based strategy is affected by pool numbers and viral loads in the samples [15,16,17]. Therefore, the evaluation of sample pooling for specific test and sample types is needed.

In addition to the main respiratory problem, SARS-CoV-2 also causes gastrointestinal symptoms in both humans and animals. It was reported that SARS-CoV-2 was detected in different types of clinical specimens with variable rates including blood (1%), feces (29%), pharyngeal swabs (32%), fibrobronchoscope brush biopsies (46%), sputum (72%), nasal swabs (63%), and bronchoalveolar lavage fluid (93%) [18]. Viral loads in different types of samples may change during the progression of the disease. One study revealed that 53 of 127 patients who tested positive for SARS-CoV-2 via a nasopharyngeal swab concurrently produced a positive result when their stools were tested. However, 31 of 84 patients who tested negative in their nasopharyngeal swabs were tested positive in stools [19]. On the veterinary side, the University of Illinois Veterinary Diagnostic Laboratory (UI-VDL) took advantage of using animal feces as a testing specimen, as this can be a non-invasive and convenient sample to collect from most animal populations. Using this protocol, we are able to successfully detect SARS-CoV-2 by real-time reverse transcriptase polymerase chain reaction (rRT-PCR) in different animal species including tigers, lions, snow leopards, gorillas, coatimundis, fishing cats, binturongs, and mandrills [20,21]. The use of this sample type is further supported by data that indicate non-variant SARS-CoV-2 shedding in feces can range from 12 to 26 days in tigers, 16–39 days in lions, and 30–33 days in snow leopards [22,23], while Delta variant shedding in feces has been noted to last 11 and 19 days in two snow leopards with clinical signs but only 5 days in an asymptomatic snow leopard, and 14–20 days in lions [20]. Therefore, the shedding periods of SARS-CoV-2 in animals could be affected by disease severity, animal species, and strain virulence. To scale up testing capacity for SARS-CoV-2 in animals, the evaluation of sample pooling for detection in animals is essential.

Furthermore, as the immunity of populations has increased over the past four years, detailing a sensitive and reliable method to detect SARS-CoV-2 in subclinical animals, or those with minimal signs, is of increasing importance. Timely diagnostic results for large numbers of individuals, amidst the other demands which occur during such large-scale events, can be affected by delays in shipping, processing, and testing. These potentials make it vital to determine the stability of the genome and appropriately characterize the reliability of test results under various conditions which may arise.

Thus, in the present study, we aim to evaluate the utility of fecal sample pooling for use in the testing of SARS-CoV-2 in animals and to assess pre-analytical variables associated with sample storage that could impact results. Our findings will inform an appropriate methodology for enhancing diagnostic detection.

## 2. Materials and Methods

### 2.1. Viral Isolates and Banked Clinical Samples

Gamma-Irradiated SARS-CoV-2 isolate USA-WA1/2020, (BEI resources catalog No. NR-52287) was used in the measurement of the limit of detection (LOD) for 5-sample and 10-sample pooling. Heat-inactivated SARS-CoV-2 isolate 2019-nCoV/USA-WA1/2020 (ATCC catalog # VR-1986HK) and Omicron variant hCoV-19/USA/GA-EHC-2811C/2021 (BEI resources catalog No. NR-56495) were used in the blinded method test (BMT). The genome copy number of these isolates was determined using the BioRad QX200 Droplet Digital PCR (ddPCR™) System by BEI resources (Bio-Rad Laboratories, Inc., Hercules, CA, USA).

Banked fecal samples of zoo animals previously confirmed positive (all from tigers and lions, most of them with clinical signs) or negative (many different captive species including lions, leopards, gorillas, etc.) by SARS-CoV-2 rRT-PCR were used to evaluate the effect of 5-sample and 10-sample pooling on SARS-CoV-2 real-time RT-PCR testing, the simulation of 5-sample pooling, and the stability of a SARS-CoV-2 positive fecal PBS suspension solution.

### 2.2. Extraction of Nucleic Acid and rRT-PCR

The nucleic acids of individual and pooled samples were extracted using the MagMAX™ Pathogen RNA/DNA Kit on a KingFisher Flex machine (ThermoFisher, Waltham, MA, USA). An amount of 200 µL of the sample for extraction and 60 µL of an elution buffer was used.

rRT-PCR was performed on a Bio-Rad CFX Opus 96 Real-Time PCR instrument (Bio-Rad Laboratories, Inc., Hercules, CA, USA) and Bio-Rad CFX Maestro software 2.3 using the CDC-based N1 primers and probe for SARS-CoV-2 [24]. The AgPath-ID™ One-Step RT-PCR kit was used in the assay. A 1× master mix recipe consisted of 2.5 µL biological grade water, 12.5 µL 2× buffer, 2 µL COVID-19 N gene primer/probe, 1 µL LIZ assay, 1 µL Xeno, and 1 µL 25× RT-PCR enzyme. Each reaction contained 20 µL of master mix and 5 µL of the extracted sample. One Negative Template Control (Molecular Biological Grade Water, Corning, Glendale, AZ, USA) and one Positive Amplification Control were included for each PCR. Upon the completion of the run, fluorescence thresholds were analyzed to ensure the Ct values were appropriately recorded.

For the stability of SARS-CoV-2 RNA in fecal PBS suspensions, in addition to the AgPath-ID One-Step kit for the N1 region, the TaqPath™ COVID-19 Combo Kit (ThermoFisher, Waltham, MA, USA) for three different regions (ORF1ab, S, and N) was also used following the kit’s instruction.

### 2.3. LOD Measurement of 5-Sample and 10-Sample Fecal Pools

The USA-WA1/2020 strain was 10-fold diluted (6.4 × 10^5^ to 6.4 genome copies) and 2-fold diluted (6.4, 3.2, and 1.6 genome copies) then spiked into 500 µL fecal PBS suspensions. SARS-CoV-2 negative feces of large cat feces were used to prepare the fecal PBS suspensions. After a 15 s vortex and a two-minute centrifugation at 8000 rpm of the spiked fecal PBS solution and negative fecal PBS solutions, the following were used for the extraction of nucleic acid. For the 5-sample pools, 40 µL of the supernatant of spiked samples and 160 µL of negative fecal PBS were used. For the 10-sample pools, 20 µL of the supernatant of spiked samples and 180 µL of negative fecal PBS were used. An amount of 200 µL of the originally spiked samples was also used for nucleic acid extraction. Both the extraction and the RT-PCR were performed in duplicate.

### 2.4. Evaluation of Effect of 5-Sample and 10-Sample Fecal Pooling on SARS-CoV-2 rRT-PCR

For the five-sample pool, five fecal specimens containing one known SARS-CoV-2 positive combined with four known SARS-CoV-2 negatives were equally (40 µL each) combined. For the ten-sample pool, ten fecal specimens with one known SARS-CoV-2 positive and nine known SARS-CoV-2 negatives were equally (20 µL each) combined. In total, 49 SARS-CoV-2 positive fecal samples were swabbed into 1000 µL PBS and evaluated in both 5-sample and 10-sample pools. The amalgamated samples along with the individual positive samples were processed for the extraction of nucleic acids and rRT-PCR.

### 2.5. Simulation of 5-Sample Pooling

A total of 30 simulated 5-sample pools were evaluated using 29 SARS-CoV-2 positive and 121 SARS-CoV-2 negative fecal samples. Each of these 150 fecal samples was swabbed into an Eppendorf tube with 1000 µL PBS followed by a 15 s vortex. All tubes with fecal PBS suspension were then randomly mixed in a container and sorted into 30 groups of five samples each, performed by another laboratory member. Each tube within each pool was then centrifuged for 2 min at 8000 rpm, and 40 µL of supernatant in each tube was used for pooling. The mixtures and individual samples were used for the extraction of nucleic acids and rRT-PCR.

### 2.6. BMT of 5-Sample Pools

BMT is an exercise in which an independent laboratory prepares samples and ships them to the method-originating laboratory for blinded (i.e., unbiased) analysis. BMT samples were prepared by spiking inactivated viral isolates (e.g., 2019-nCoV/USA-WA1/2020 and hCoV-19/USA/GA-EHC-2811C/2021) into feline fecal homogenate (i.e., a mixture of feces in PBS; ~12% by weight) or 1× PBS. The fecal homogenate was prepared by swabbing feces with cotton tipped applicators, placing them into PBS (1 swab per 1 mL), vortexing, and pre-aliquoting the mixture into 1.5 mL tubes. More details can be found in the online protocol [25]. A total of 352 tubes were pre-aliquoted with 176 designated for the fecal matrix and 176 tubes for PBS. Of the 176 tubes for each matrix (feces or PBS), 36 tubes were filled with 450 µL of the fecal matrix or PBS alone and spiked with 50 µL of diluted heat inactivated virus or PBS to create 500 µL test samples. The remaining 140 tubes for each matrix (feces or PBS) were filled with 500 µL of either PBS or the fecal matrix only and were used as negative samples for pooling. For each matrix (feces and PBS), the FDA Veterinary Laboratory Investigation and Response Network (Vet-LIRN) assigned samples to 64 groups of five samples per group, with each group containing one test sample and four negative samples (Table S1). All samples were kept on ice during spiking and samples were shipped overnight to the UI VDL on dry ice and stored at −80 °C until extraction was performed. Following the BMT’s instruction, each sample was vortexed, centrifuged at 8000 rpm for 2 min, and pooled using 40 µL from each tube. Pooled samples were extracted and analyzed by rRT-PCR.

### 2.7. Stability of SARS-CoV-2 Genome in Positive Fecal PBS Suspensions

To evaluate the stability of SARS-CoV-2 RNA in feces, three clinical positive samples were chosen and sixteen cotton-tipped swabs, one at a time, were used to collect fecal material from each sample and subsequently placed into 19 mL of PBS. The final solution was vortexed and 300 µL was aliquoted into fifty-eight 1.5 mL Eppendorf microcentrifuge tubes. Aliquots were then assigned one of twenty-eight different time and temperature conditions under which to be maintained. Storage conditions consisted of four different temperatures (room temperature, 4 °C, −20 °C, and −80 °C) across seven different time frames (1 day, 3 days, 5 days, 7 days, 10 days, 14 days, and 28 days). One set of samples was immediately extracted (day zero) and the RNA content evaluated for use as a Ct reference value. Each set of parameters contained two samples to be assessed in duplicate. To minimize variations associated with processing and measurement, −80 °C samples were stored together and RNA was extracted at the appropriate time points, progressing from day zero, whereas the room temperature, 4 °C, and −20 °C samples were placed in their respective environments in reverse order, beginning with day twenty-eight and progressing to day one. This allowed the latter set of samples to undergo RNA extraction at the same time. Finally, all sample RNA content was evaluated by rRT-PCR in a single run. Ct value differences for each time point at different temperatures were determined by subtracting them from the Ct of the samples at day 0.

## 3. Results

### 3.1. LOD Measurement of 5-Sample Pooling and 10-Sample Pooling

Real-time RT-PCR results showed that SARS-CoV-2 can be detected in 5-sample fecal pools ranging from 1.28 × 10^5^ down to as low as 12.8 genome copies, while 6.4 × 10^4^ to 6.4 genome copies of SARS-CoV-2 can be detected in 10-sample fecal pools (Figure S1). Linear regression analysis revealed correlation coefficients of R^2^ = 0.999 for both 5- and 10-sample fecal pool testing. The LODs of both 5- and 10-sample pooling measured in our study were close to the five genome copies reported previously [24].

### 3.2. Effect of 5-Sample and 10-Sample Fecal Pooling on SARS-CoV-2 Real-Time RT-PCR

A total of forty-nine SARS-CoV-2 positive samples, with Ct values ranging from 17.05 to 39.65, were evaluated in five-sample and ten-sample pools by combining them individually with four and nine SARS-CoV-2 negative fecal samples, respectively. The results showed that by combining specimens into pools of 5 and 10 samples, the Ct value increased by 2.35 and 3.45, respectively. Regardless, we could consistently detect all positives in both 5- and 10-sample pools with Ct values below 36 and 33.7, respectively, in the original unpooled samples (Table 1). For those samples with original Ct values above 36 and above 33.7, respectively, 5-sample and 10-sample pooling might miss the detection of SARS-CoV-2 in 77% (10/13) and 76% (13/17) of cases, respectively.

### 3.3. Simulation of 5-Sample Pooling

Randomly mixed 121 SARS-CoV-2 negative and 29 SARS-CoV-2 positive samples produced thirty 5-sample pools. Real-time RT-PCR results revealed that 20 of the 30 contained positive samples (Table 2). Among the 20 positive pools, 13 contained only one positive, 5 had two positives, and 2 had three positives. Overall, the pools with positive samples resulted in an average Ct value increase of 2.36 (range: 1.45–3.37) (Table 2). Since more than one positive sample within those pools with two and three positives (P4, P7, and P23) had a loss of Ct value, the value associated with the loss of Ct, in the seven pools which contained more than one positive subset, was calculated based on the subset with the lowest Ct value.

### 3.4. BMT of 5-Sample Pooling

The Rate of Detection (ROD) for BMT samples was as expected for all PBS and fecal samples for both WA-1 and Omicron variants: 0% detection for all blank samples and 100% detection for high (1332 copies/reaction) and low (120 copies/reaction) samples (Table 3). The analysis from day 1 and 2 gave the same ROD at all spiking levels, between variants, and between matrixes, indicating strong reproducibility for this methodology of SARS-CoV-2 detection in pools of five samples. Ct values were compared between spike levels and the matrix. Ct values were consistent between spike levels regardless of the variant and matrix. Of note, there was a consistent Ct shift of ~2–3 Ct values between PBS and the fecal matrix (Table 4). This indicates there is a matrix effect, and this could impact detection for low viral RNA samples. There was also a consistent Ct shift between variants, with Ct values for Omicron samples being 1.5–3-fold higher than WA-1 (Table 4).

### 3.5. Stability of SARS-CoV-2 Positive Fecal PBS Suspensions

Three fecal samples, #1, 2, and 3, all positive for the SARS-CoV-2 genome (Ct values of 20, 24, and 28, respectively), were used for evaluating the stability of RNA in fecal PBS solutions. All samples stored at different temperatures and collected at various time points were analyzed at the same time. For the evaluation of Ct values, we used the average of the duplicate samples for a given time and temperature parameter.

**Figure 1:**
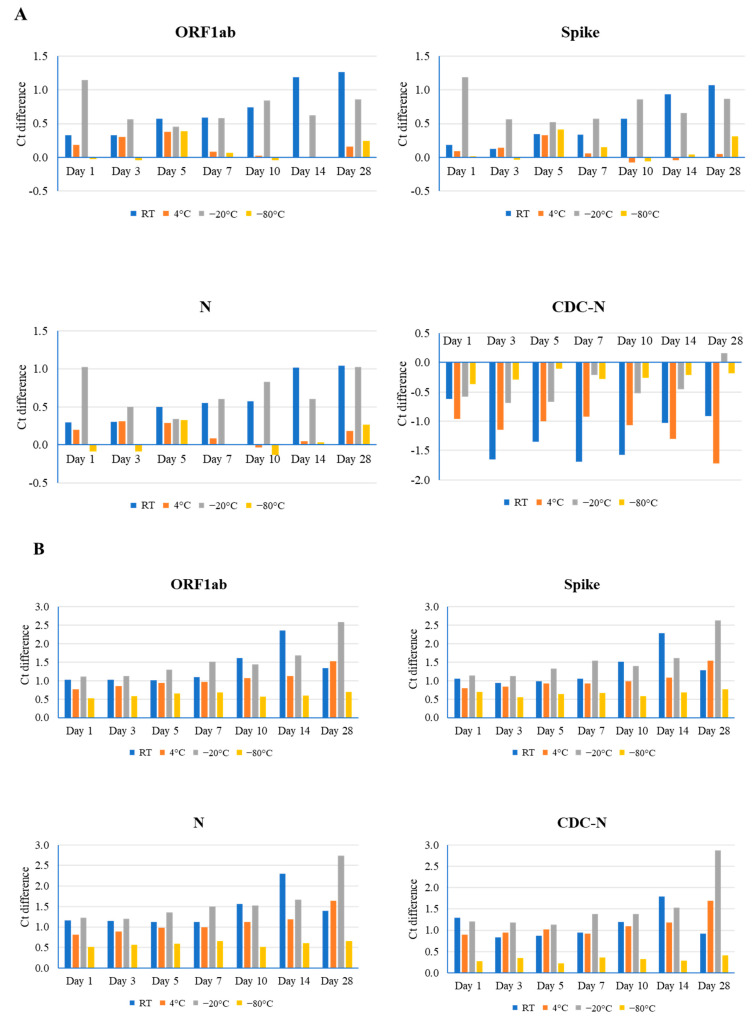

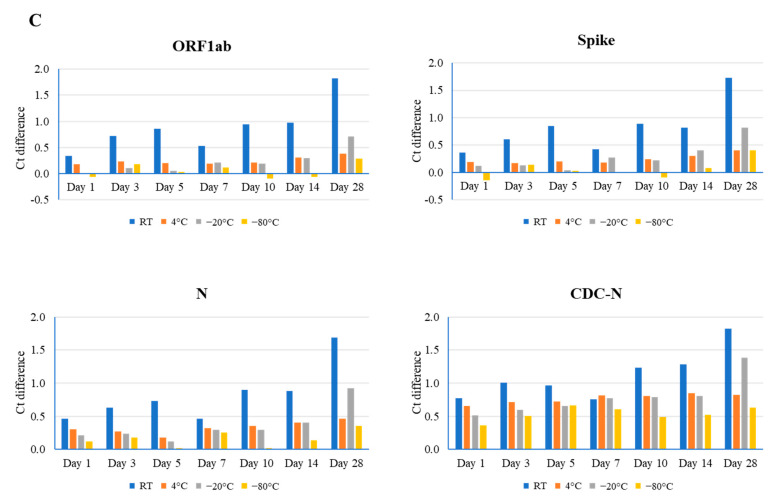
Comparison of different time point (days 1, 3, 5, 7, 10, 14, and 28) and temperature (room temperature (RT), 4 °C, −20 °C, and −80 °C) effects on the stability of the SARS-CoV-2 genome, as determined by real-time RT-PCR using the TaqPath Combo SARS-CoV-2 kit (ORF1ab, spike, and N) and CDC-based SARS-CoV-2 N1 (CDC-N). Ct difference indicates the value above or below the reference Ct at day 0 for that sample. Three clinical samples with high ((**A**), Ct value 20), medium ((**B**), Ct value 24), and low ((**C**), Ct value 28) Ct values were evaluated.

For sample #1, the lowest Ct value (20), effects of room temperature on RNA stability were observed in all three genes. A gradual increase in value was correlated with longer storage periods, and the maximal Ct value difference was observed in the ORF1ab gene (1.26) at day 28. Storage at 4 °C had minimal to no effect on gene integrity at various time points, as evidenced by the maximal effect observed in the ORF1ab gene (0.38) at day 5 (Figure 1A and Figure S2). By contrast, −20 °C had notable effects, producing a non-uniform pattern in Ct values. The maximal effect for this condition was again observed in ORF1ab (1.14), but on day 1. Unsurprisingly, samples stored at −80 °C had negligible (up to 0.41) or no effects on stability. Overall, temperature-related effects on RNA integrity were noted in genes analyzed using the TaqPath COVID-19 Combo kit (ThermoFisher, Waltham, MA, USA), but not in the CDC-based N gene. It is surmised that the relatively small effect of different temperatures on the SARS-CoV-2 RNA stability could be due to a higher amount of RNA present in this sample. Therefore, two other samples (#2 and #3) with higher Ct values were used for additional evaluation.

For sample #2, the mid-range Ct value (24), room temperature, 4 °C, and −20 °C had notable effects on RNA stability. As well as this, the largest effects were associated with the longer storage times of days 14, 28, and 28 (Ct difference: 2.36 in ORF1ab, 1.69 in the N gene, and 2.88 in the N gene, respectively). Conversely, the smallest effects occurred at days 3, 1, and 3 (Ct difference: 0.83 in the N gene, 0.76 in ORF1ab, and 1.19 in the N gene, respectively) (Figure 1B and Figure S2). Again, samples stored at −80 °C revealed a minimal effect on stability, with a range in Ct difference from 0.22 to 0.77.

For sample #3, the highest Ct value (28), storage at room temperature and −20 °C yielded greater effects on stability than at 4 °C and −80 °C, generating the largest differences in Ct value at day 28 (1.82 in ORF1ab at room temperature and 1.38 in the N gene at −20 °C). The least amount of effect in these samples was observed on day 1 (Ct difference: 0.34 in ORF1ab at room temperature and 0.01 in ORF1ab at −20 °C) (Figure 1C and Figure S2). Storage at 4 °C temperature had the least (Ct difference: 0.17 in the S gene) and greatest (Ct difference: 0.85 in the N gene) effect on gene integrity at days 3 and 14, while storage at −80 °C had no effect on stability at several time points for the ORF1ab and S genes, and the largest effect (Ct difference: 0.66 in the N gene) at day 5.

## 4. Discussion

Sample pooling has been a long-standing approach for diagnostics that provides an efficient method for the screening of large populations. In the veterinary field, this practice is routinely used for testing animal samples for different diseases in the US including porcine reproductive and respiratory syndrome (PRRS), bovine virus diarrhea (BVD), avian influenza (AI), Newcastle disease (ND), Johne’s disease, and bovine anaplasmosis. Depending on animal species and screening purposes, sample pooling occurs in two different ways: by compiling samples at the sample collection site (AI and ND), or by pooling individually submitted samples at the laboratory (PRRS, BVD, Johne’s disease, and bovine anaplasmosis). For example, pools of swabs from 5 or 11 birds can be prepared using 3 mL or 5.5 mL viral transport media, respectively, and used for AI and ND testing, as stated by the USDA [26]. By contrast, ear notch samples can be pooled in quantities up to 50 for the PCR testing of persistently infected BVD animals [27]. Given its efficacy, sample pooling was quickly implemented during the early pandemic period for scaling up SARS-CoV-2 testing in humans. For instance, the testing of 9- and 10-nasopharyngeal sample pools was performed in Spain [14]. However, sample pooling might not be an efficient approach when the SARS-CoV-2 prevalence is high, whereas it is a very useful strategy for testing when positive rates are low. A simulation of pooling study showed that when the prevalence is less than 30%, a sample pooling strategy will have higher efficiency than individual testing [28]. In general, positive rates of SARS-CoV-2 in animals such as captive animals and wildlife are relatively low and therefore sample pooling might be a useful approach to enhance testing capacity for SARS-CoV-2 in animals.

In the present study, we evaluated the effect on SARS-CoV-2 testing using 5-sample and 10-sample fecal pools, and our data demonstrated that this method could be useful for the large-scale testing of SARS-CoV-2 in animals. It has been noted that the number of samples in a pool and the specific molecular methods employed can affect the sensitivity of a SARS-CoV-2 PCR test. A previous study showed that depending on sample viral loads, four-sample nasopharyngeal swab pooling had sensitivities of 50% and 75%, whereas eight-sample pooling had lower sensitivities of 2% and 62.5% [17]. On the other hand, another study determined that 9–10 nasopharyngeal swab pooling had a sensitivity of 85.5% for SARS-CoV-2 testing [14]. In the present study, we showed that five-fecal and ten-fecal sample pooling had sensitivities of 81.6% and 73.5%, respectively (Table 1), whereas five-sample pooling simulation data revealed a much higher sensitivity of 100% (Table 2). The difference between sensitivities noted by our study is the result of a higher percentage of Ct values above 35.99 in the sampling pooling study (26.5%) as compared to the percentage tested in the simulation study (3.4%).

Our data showed that 5-fecal sample pooling could result in a Ct value loss of up to 2.34–2.35, and up to a Ct value loss of 3.45 in 10-sample pooling. In animals, respiratory samples from nasal swabs typically have a relatively higher SARS-CoV-2 viral load than feces (unpublished data). However, fecal material is an ideal sample as it can be a non-invasive and convenient sample to collect, especially from wild animals and those in captivity. To avoid losing a significant amount of sensitivity, the pooling of up to five samples is recommended for large-scale surveillance.

To determine the ROD for five-fecal sample pooling, a BMT was conducted in collaboration with Vet-LIRN. The BMT protocol included two SARS-CoV-2 strains, Omicron and non-variant WA-1, placed in matrixes of either feces or PBS. The ROD for BMT samples was as expected for all PBS and fecal samples for both WA-1 and Omicron variants; however, a matrix effect was observed with a shift in Ct value between the fecal matrix and PBS. Biomatrices like feces contain microbial communities and enzymes that can break down pathogens, altering detection [29]. Therefore, the effect seen here is not unexpected or concerning, as even low-level spiked samples were 100% detected in both PBS and the fecal matrix, indicating that this is a reliable method of detection even from fecal samples. Furthermore, PBS is a highly suitable medium in which to conduct these tests [30]. If other biomatrices or storage media are to be used for screening by this method, it is recommended that additional validation be conducted.

While our data support the pooling of fecal samples for clinical use, we recognize the fact that this methodology is commonly employed during times when surge testing is needed. In these instances, the risk of delay associated with shipping, processing, and testing is increased. To this end, both clinicians and diagnostic labs must have some notion as to the reliability of the results when samples succumb to less than optimum conditions. Our results show that the difference between each time/temperature condition and their respective reference Ct value ranged from 0.00 to 2.88. Ct values with a difference greater than 0.8 from their respective reference occurred infrequently in samples 1 (two time points of room temperature and three time points of −20 °C) and 3 (only for three–five time points of room temperature). Interestingly, sample 2 (Ct reference of 24) yielded the most variation. All the samples stored at room temperature, 4 °C (except day 1 in ORF1ab), and −20 °C resulted in Ct differences greater than 0.8. Given the data as a whole, there is no apparent reason for this anomaly. It should also be noted that the −20 °C freezer did not have a freeze–thaw cycle, and therefore variation in temperature was not a factor for this condition. Based on our sample stability findings, it is suggested that the exposure of fecal samples to room temperature should be minimized, and they should only be stored in refrigerators for a few days. Ideally, long-term storage in −80 °C freezers, instead of −20 °C, should be the standard practice.

In summary, our study evaluated 5- and 10-fecal sample pooling for SARS-CoV-2 real-time RT-PCR testing in animals. Our data demonstrated that the sensitivities of sample pooling were affected by both the numbers of pooled samples and the viral loads of samples. We also observed the effect of the fecal matrix on the detection of the SARS-CoV-2 genome. Our data support pooling up to five fecal samples for the real-time PCR testing of SARS-CoV-2, without sacrificing a significant level of sensitivity. In conclusion, we evaluated this method and showed that the fecal sample pooling method could be useful for SARS-CoV-2 surveillance in animals in future.

## Figures and Tables

**Table 1:** Effect of 5-sample (5-pool) and 10-sample (10-pool) pooling on real-time RT-PCR for SARS-CoV-2. Ct values of original non-pooled 49 individual samples, 5-pool, and 10-pool are listed. Ct loss of 5-pool and 10-pool compared with corresponding individuals is calculated.

Sample #	Individual	5-Pool	10-Pool	5-Pool Ct Loss	10-Pool Ct Loss
S1	17.05	19.42	20.46	2.37	3.41
S2	18.66	21.38	22.44	2.72	3.78
S3	18.88	21.44	22.41	2.56	3.54
S4	20.71	23.23	24.27	2.52	3.55
S5	21.54	24.06	25.06	2.52	3.52
S6	22.25	25.15	26.01	2.90	3.75
S7	22.50	24.85	25.82	2.34	3.32
S8	23.64	26.48	27.41	2.84	3.77
S9	23.73	26.14	27.16	2.41	3.44
S10	24.67	27.45	28.31	2.78	3.64
S11	24.90	27.19	28.35	2.28	3.45
S12	24.94	27.66	28.80	2.71	3.85
S13	27.55	29.78	31.33	2.23	3.77
S14	28.05	30.49	31.78	2.44	3.73
S15	28.29	30.84	32.06	2.54	3.77
S16	28.41	31.02	32.4	2.61	3.98
S17	28.49	30.75	31.66	2.26	3.17
S18	28.59	30.72	31.81	2.13	3.21
S19	29.15	31.42	32.66	2.27	3.51
S20	30.49	32.53	34.08	2.04	3.59
S21	30.66	32.70	33.64	2.04	2.98
S22	31.16	33.7	35.42	2.54	4.26
S23	31.21	33.88	34.62	2.67	3.42
S24	31.30	34.18	34.78	2.88	3.49
S25	31.41	33.45	34.51	2.04	3.10
S26	32.00	34.80	34.91	2.80	2.91
S27	32.12	34.43	37.77	2.32	5.66
S28	32.34	34.62	35.87	2.28	3.53
S29	32.4	34.55	35.3	2.14	2.89
S30	33.27	36.16	35.81	2.89	2.53
S31	33.66	35.40	37.13	1.75	3.47
S32	33.66	35.66	37.1	2	3.44
S33	33.80	37.84	ND	4.04	-
S34	35.02	37	37.65	1.98	2.63
S35	35.25	37.79	ND	2.54	-
S36	35.34	37.48	38.58	2.15	3.25
S37	36.03	ND	38.82	-	2.79
S38	36.04	37.03	ND	1	-
S39	36.06	37.1	38.14	1.04	2.08
S40	37.63	38.51	ND	0.88	-
S41	36.50	ND	ND	-	-
S42	37.09	ND	ND	-	-
S43	37.14	ND	ND	-	-
S44	37.16	ND	ND	-	-
S45	37.61	ND	ND	-	-
S46	37.79	ND	ND	-	-
S47	38.73	ND	ND	-	-
S48	39.36	ND	ND	-	-
S49	39.65	ND	ND	-	-
Average Ct value loss				2.35	3.45

ND: not detectable. -: not applicable.

**Table 2:** Simulation of 5-sample pooling using 121 SARS-CoV-2 negative and 29 SARS-CoV-2 positive samples. Twenty 5-sample pools with 1–3 positive samples were identified, and Ct values of all positive pools were compared with those of the individual positive samples.

5-Pool#	Pool Ct	Individual Ct					Ct Loss
P1	28.75	26.20	-	-	-	-	2.55
P2	32.11	29.65	-	-	-	-	2.45
P3	22.03	19.60	-	-	-	-	2.44
P4 *	34.90	33.01	34.10	-	-	-	1.89
P5	0.00	-	-	-	-	-	
P6	0.00	-	-	-	-	-	
P7 ^&^	33.59	31.81	34.74	32.72	-	-	1.78
P8	33.55	31.27	-	-	-	-	2.29
P9	30.17	27.55	-	-	-	-	2.62
P10	27.70	25.33	-	-	-	-	2.37
P11	0.00	-	-	-	-	-	
P12 *	30.15	27.75	31.67	-	-	-	2.39
P13	0.00	-	-	-	-	-	
P14	0.00	-	-	-	-	-	
P15	38.66	37.21	-	-	-	-	1.45
P16	34.12	31.71	-	-	-	-	2.41
P17	0.00	-	-	-	-	-	
P18 *	25.32	23.13	30.67	-	-	-	2.19
P19	37.02	34.10	-	-	-	-	2.92
P20 ^&^	29.97	27.44	31.94	30.75	-	-	2.52
P21	0.00	-	-	-	-	-	
P22	0.00	-	-	-	-	-	
P23 *	23.09	21.63	22.17	-	-	-	1.46
P24	29.78	27.31	-	-	-	-	2.47
P25	0.00	-	-	-	-	-	
P26	35.07	31.69	-	-	-	-	3.37
P27	32.05	28.92	-	-	-	-	3.13
P28	0.00	-	-	-	-	-	
P29 *	25.71	23.11	33.55	-	-	-	2.61
P30	19.47	17.54	-	-	-	-	1.93
	Average Ct loss						2.36

-: no Ct value for all these SARS-CoV-2 negative samples. *: pools with two positive samples; Ct loss was determined by the samples with the lowest Ct value. ^&^: pools with three positive samples; Ct loss was determined by the samples with the lowest Ct value.

**Table 3:** Rate of Detection (ROD) of the blinded method test (BMT) with low (120 copies/reaction) and high (1332 copies/reaction) spiked SARS-CoV-2 WA-1 (2019-nCoV/USA-WA1/2020) and Omicron (hCoV-19/USA/GA-EHC-2811C/2021) in the PBS and fecal matrixes.

			Day 1		Day 2		Total
Matrix	Variant	Spike Levels	# False Positives	# False Negatives	# False Positives	# False Negatives	ROD
PBS	WA-1	Blank	0/2	-	0/2	-	0/4
		High	-	0/3	-	0/3	6/6
		Low	-	0/3	-	0/3	6/6
	Omicron	Blank	0/2	-	0/2	-	0/4
		High	-	0/3	-	0/3	6/6
		Low	-	0/3	-	0/3	6/6
Fecal	WA-1	Blank	0/2	-	0/2	-	0/4
		High	-	0/3	-	0/3	6/6
		Low	-	0/3	-	0/3	6/6
	Omicron	Blank	0/2	-	0/2	-	0/4
		High	-	0/3	-	0/3	6/6
		Low	-	0/3	-	0/3	6/6

**Table 4:** Day 1 and Day 2 sample results from the UI-VDL with key. Low and high spiked concentrations of SARS-CoV-2 WA-1 (2019-nCoV/USA-WA1/2020) and Omicron (hCoV-19/USA/GA-EHC-2811C/202) are 120 and 1332 copies/reaction, respectively. Detailed information on all groups is available in Table S1.

Sample Day 1								
Feces Group	Feces Test Samples	Ct	Average Ct	PBS Groups	PBS Test Samples	Ct	Average Ct	Ct Difference: Feces vs. PBS
Group-2	WA-1 Blank	0.00		Group-33	WA-1 Blank	0.00		
Group-5	WA-1 Blank	0.00		Group-37	WA-1 Blank	0.00		
Group-1	WA-1 Low	37.83	37.5	Group-34	WA-1 Low	33.50	33.7	3.81
Group-4	WA-1 Low	37.32		Group-35	WA-1 Low	34.03		
Group-6	WA-1 Low	37.61		Group-39	WA-1 Low	33.79		
Group-3	WA-1 High	34.83	34.8	Group-36	WA-1 High	31.33	30.8	3.99
Group-7	WA-1 High	34.48		Group-38	WA-1 High	30.66		
Group-8	WA-1 High	35.29		Group-40	WA-1 High	30.65		
Group-10	Omicron—Blank	0.00		Group-42	Omicron—Blank	0.00		
Group-13	Omicron—Blank	0.00		Group-45	Omicron—Blank	0.00		
Group-9	Omicron—Low	37.18	36.8	Group-41	Omicron—Low	34.26	34.5	2.32
Group-12	Omicron—Low	36.98		Group-44	Omicron—Low	34.62		
Group-15	Omicron—Low	36.47		Group-47	Omicron—Low	34.79		
Group-11	Omicron—High	32.46	32.6	Group-43	Omicron—High	31.13	31.0	1.67
Group-14	Omicron—High	32.82		Group-46	Omicron—High	30.70		
Group-16	Omicron—High	32.81		Group-48	Omicron—High	31.25		
**Sample Day 2**								
**Feces Group**	**Feces Test Samples**	**Ct**	**Average Ct**	**PBS**	**PBS**	**Ct**	**Average Ct**	**Ct Difference: Feces vs. PBS**
Group-17	WA-1 Blank	0.00		Group-50	WA-1 Blank	0.00		
Group-22	WA-1 Blank	0.00		Group-51	WA-1 Blank	0.00		
Group-19	WA-1 Low	36.13	35.5	Group-54	WA-1 Low	32.55	32.5	2.93
Group-20	WA-1 Low	35.35		Group-52	WA-1 Low	32.89		
Group-23	WA-1 Low	35.06		Group-56	WA-1 Low	32.31		
Group-18	WA-1 High	33.12	32.8	Group-49	WA-1 High	29.80	29.6	3.19
Group-21	WA-1 High	32.80		Group-53	WA-1 High	29.54		
Group-24	WA-1 High	32.69		Group-55	WA-1 High	29.70		
Group-25	Omicron—Blank	0.00		Group-57	Omicron—Blank	0.00		
Group-26	Omicron—Blank	0.00		Group-58	Omicron—Blank	0.00		
Group-27	Omicron—Low	36.31	36.2	Group-59	Omicron—Low	33.07	33.4	2.80
Group-28	Omicron—Low	35.68		Group-60	Omicron—Low	33.72		
Group-30	Omicron—Low	36.78		Group-62	Omicron—Low	33.77		
Group-29	Omicron—High	32.57	32.2	Group-61	Omicron—High	30.37	30.21	1.98
Group-31	Omicron—High	31.69		Group-63	Omicron—High	30.34		
Group-32	Omicron—High	32.52		Group-64	Omicron—High	29.93		

## Data Availability

The authors confirm that the data supporting the findings of this study are available within the article; additional data related to this study are available from the corresponding author upon reasonable request.

## Supplementary Materials

The following supporting information can be downloaded at https://www.mdpi.com/article/10.3390/v16111651/s1: Figure S1: Limit of detection of 5-sample and 10-sample pooling on SARS-CoV-2 real-time RT-PCR; Figure S2: Average Ct difference in all three samples (their original Ct values are 20, 24, and 28) at different time points (days 1, 3, 5, 7, 10, 14, and 28) and temperatures (room temperature (RT), 4 °C, −20 °C, and −80 °C); Table S1: Vet-LIRN-assigned groups for analysis by UI VDL.
