# Supplementary material for: Evaluation of Fecal Sample Pooling for Real-Time RT-PCR Testing SARS-CoV-2 in Animals
Source: Viruses. 2024 Oct 23;16(11):1651. doi: 10.3390/v16111651 (PMC11599033; doi:10.3390/v16111651)
Supplement: Supplementary file 1 [file viruses-16-01651-s001.zip › viruses-3183441-supplementary.pdf]

Supplemental files

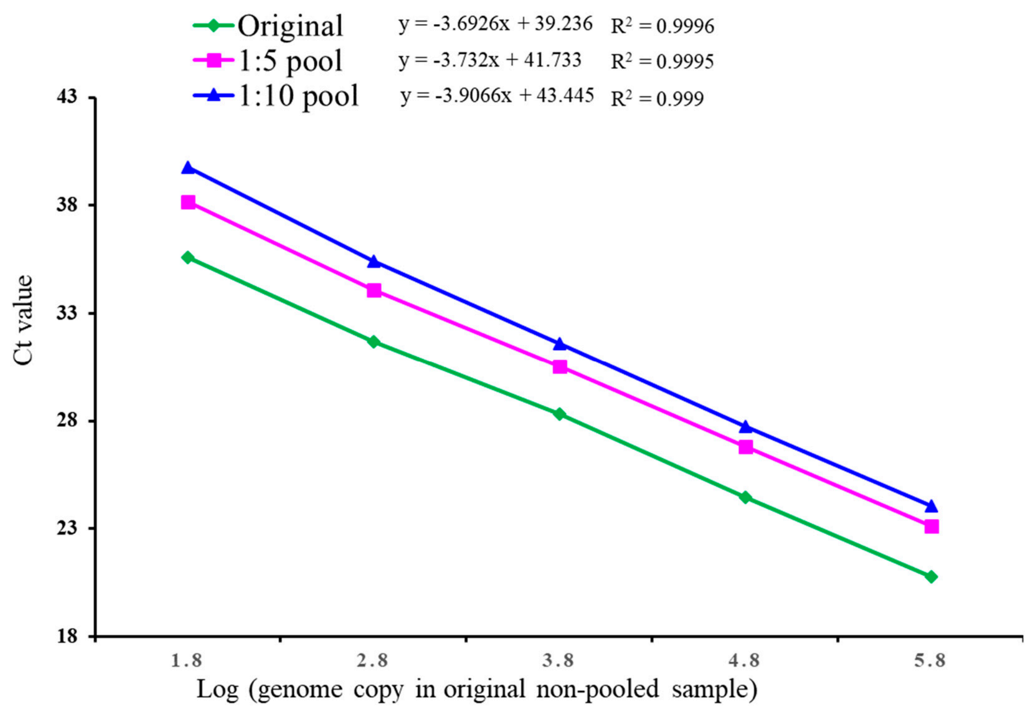

Figure S1. Limit of detection of 5-sample and 10-sample pooling on SARS-CoV-2 real-time RT-PCR.

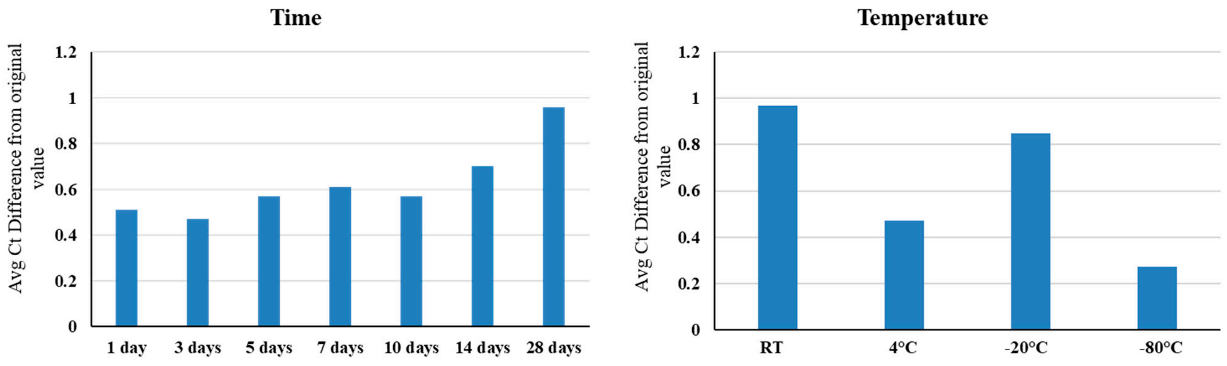

Figure S2. Average Ct difference of all three samples (their original Ct values are 20, 24, 28) at different time points (Day 1, 3, 5, 7, 10, 14, and 28) and temperatures (room temperature (RT), 4°C, -20°C and -80°C).

Table S1: Vet-LIRN assigned groups for analysis by UI VDL

Group #	sample ID#	tube #	Day of Analysis	content	Group #	sample ID#	tube #	Day of Analysis	content
1	90	1	1	Blank (feces)		129	1	1	Blank (PBS)
	21	2	1	Blank (feces)	33	152	2	1	Blank (feces)
	52	3	1	Blank (feces)		8	3	1	Blank (PBS)
	136	4	1	Blank (feces)		109	4	1	Blank (PBS)
	114	5	1	Low		76	5	1	Blank (PBS)
2	15	6	1	Blank (feces)		147	6	1	Blank (PBS)
	91	7	1	Blank (feces)		46	7	1	Blank (PBS)
	81	8	1	Blank (feces)		146	8	1	Blank (PBS)
	123	9	1	Blank (feces)	34	92	9	1	Blank (feces)
	93	10	1	Blank (feces)		6	10	1	Low
3	16	11	1	Blank (feces)		59	11	1	Blank (PBS)
	98	12	1	Blank (feces)		53	12	1	Blank (PBS)
	63	13	1	Blank (feces)	35	74	13	1	Blank (PBS)
	119	14	1	Blank (feces)		70	14	1	Blank (PBS)
	153	15	1	High		2	15	1	Low
4	50	16	1	Blank (feces)		131	16	1	Blank (PBS)
	40	17	1	Blank (feces)		134	17	1	Blank (PBS)
	99	18	1	Blank (feces)		57	18	1	Blank (PBS)
	10	19	1	Blank (feces)	36	100	19	1	Blank (PBS)
	120	20	1	Low		132	20	1	High
5	155	21	1	Blank (feces)		94	21	1	Blank (PBS)
	9	22	1	Blank (feces)		64	22	1	Blank (PBS)
	82	23	1	Blank (feces)		47	23	1	Blank (PBS)
	72	24	1	Blank (feces)	37	15	24	1	Blank (PBS)
	73	25	1	Blank (feces)		139	25	1	Blank (PBS)
6	58	26	1	Mid spike level		21	26	1	Blank (PBS)
	147	27	1	Blank (feces)		31	27	1	Blank (PBS)
	160	28	1	Blank (feces)		116	28	1	Blank (PBS)
	14	29	1	Blank (feces)	38	19	29	1	Blank (PBS)
	57	30	1	Low		133	30	1	High
7	32	31	1	Blank (feces)		117	31	1	Blank (PBS)
	70	32	1	Blank (feces)		67	32	1	Blank (PBS)
	7	33	1	Blank (feces)	39	93	33	1	Blank (PBS)
	20	34	1	Blank (feces)		12	34	1	Blank (PBS)
	131	35	1	High		34	35	1	Low
8	88	36	1	Blank (feces)		55	36	1	Blank (PBS)

	45	37	1	Blank (feces)		148	37	1	Blank (PBS)
	39	38	1	Blank (feces)	40	112	38	1	Blank (PBS)
	24	39	1	Blank (feces)		42	39	1	Blank (PBS)
	47	40	1	High		58	40	1	High
9	1	41	1	Blank (feces)		14	41	1	Blank (PBS)
	31	42	1	Blank (feces)		72	42	1	Blank (PBS)
	134	43	1	Blank (feces)	41	39	43	1	Blank (PBS)
	137	44	1	Blank (feces)		85	44	1	Blank (PBS)
	128	45	1	Low		16	45	1	Low
10	105	46	1	Blank (feces)		122	46	1	Blank (PBS)
	44	47	1	Blank (feces)		125	47	1	Blank (PBS)
	46	48	1	Blank (feces)		43	48	1	Blank (PBS)
	18	49	1	Blank (feces)	42	32	49	1	Blank (PBS)
	69	50	1	Blank (feces)		82	50	1	Blank (feces)
11	129	51	1	Blank (feces)		157	51	1	Blank (PBS)
	156	52	1	Blank (feces)		144	52	1	Blank (PBS)
	132	53	1	Blank (feces)		121	53	1	Blank (PBS)
	125	54	1	Blank (feces)	43	142	54	1	Blank (PBS)
	152	55	1	High		158	55	1	High
12	145	56	1	Blank (feces)		155	56	1	Blank (PBS)
	41	57	1	Blank (feces)		81	57	1	Blank (PBS)
	8	58	1	Blank (feces)	44	35	58	1	Blank (PBS)
	144	59	1	Blank (feces)		145	59	1	Blank (PBS)
	111	60	1	Low		25	60	1	Low
13	122	61	1	Blank (feces)		104	61	1	Blank (PBS)
	27	62	1	Blank (feces)		50	62	1	Blank (PBS)
	77	63	1	Blank (feces)	45	20	63	1	Blank (PBS)
	34	64	1	Blank (feces)		66	64	1	Blank (PBS)
	142	65	1	Blank (feces)		68	65	1	Blank (feces)
14	94	66	1	Blank (feces)		123	66	1	Blank (PBS)
	56	67	1	Blank (feces)		54	67	1	Blank (PBS)
	62	68	1	Blank (feces)	46	95	68	1	Blank (PBS)
	25	69	1	Blank (feces)		88	69	1	Blank (PBS)
	113	70	1	High		106	70	1	High
15	68	71	1	Blank (feces)		156	71	1	Blank (PBS)
	118	72	1	Blank (feces)	47	89	72	1	Blank (PBS)
	6	73	1	Blank (feces)		128	73	1	Blank (PBS)
	29	74	1	Blank (feces)		90	74	1	Blank (PBS)
	19	75	1	Low		69	75	1	Low
16	101	76	1	Blank (feces)		65	76	1	Blank (PBS)

	33	77	1	Blank (feces)		115	77	1	Blank (PBS)
	141	78	1	Blank (feces)	48	160	78	1	Blank (PBS)
	37	79	1	Blank (feces)		119	79	1	Blank (PBS)
	107	80	1	High		118	80	1	High
17	67	81	2	Blank (feces)		75	81	2	Blank (PBS)
	87	82	2	Blank (feces)		103	82	2	Blank (PBS)
	110	83	2	Blank (feces)	49	77	83	2	Blank (PBS)
	157	84	2	Blank (feces)		141	84	2	Blank (PBS)
	146	85	2	Blank (feces)		56	85	2	High
18	78	86	2	Blank (feces)		51	86	2	Blank (PBS)
	95	87	2	Blank (feces)		159	87	2	Blank (PBS)
	54	88	2	Blank (feces)	50	153	88	2	Blank (PBS)
	76	89	2	Blank (feces)		40	89	2	Blank (PBS)
	26	90	2	High		5	90	2	Blank (PBS)
19	59	91	2	Blank (feces)		83	91	2	Blank (PBS)
	11	92	2	Blank (feces)		37	92	2	Blank (PBS)
	126	93	2	Blank (feces)	51	62	93	2	Blank (PBS)
	79	94	2	Blank (feces)		97	94	2	Blank (PBS)
	130	95	2	Low		126	95	2	Blank (PBS)
20	158	96	2	Blank (feces)		114	96	2	Blank (PBS)
	151	97	2	Blank (feces)		84	97	2	Blank (PBS)
	30	98	2	Blank (feces)	52	124	98	2	Blank (PBS)
	51	99	2	Blank (feces)		48	99	2	Blank (PBS)
	143	100	2	Low		87	100	2	Low
21	127	101	2	Blank (feces)		7	101	2	Blank (PBS)
	135	102	2	Blank (feces)		136	102	2	Blank (PBS)
	2	103	2	Blank (feces)	53	143	103	2	Blank (PBS)
	55	104	2	Blank (feces)		10	104	2	Blank (PBS)
	38	105	2	High		91	105	2	High
22	138	106	2	blank (feces)		17	106	2	Blank (PBS)
	12	107	2	Blank (feces)		1	107	2	Blank (PBS)
	83	108	2	Blank (feces)	54	29	108	2	Blank (PBS)
	17	109	2	Blank (feces)		45	109	2	Blank (PBS)
	109	110	2	Blank (feces)		11	110	2	Low
23	22	111	2	Blank (feces)		49	111	2	Blank (PBS)
	124	112	2	Blank (feces)		101	112	2	Blank (PBS)
	96	113	2	Blank (feces)	55	28	113	2	Blank (PBS)
	149	114	2	Blank (feces)		52	114	2	Blank (PBS)
	61	115	2	Low		30	115	2	High
24	28	116	2	Blank (feces)		113	116	2	Blank (PBS)

	86	117	2	Blank (feces)		3	117	2	Blank (PBS)
	148	118	2	Blank (feces)	56	110	118	2	Blank (PBS)
	92	119	2	Blank (feces)		151	119	2	Blank (PBS)
	65	120	2	High		71	120	2	Low
25	5	121	2	Blank (feces)		27	121	2	Blank (PBS)
	159	122	2	Blank (feces)		44	122	2	Blank (PBS)
	43	123	2	Blank (feces)	57	138	123	2	Blank (PBS)
	66	124	2	Blank (feces)		96	124	2	Blank (PBS)
	117	125	2	Blank (feces)		60	125	2	Blank (PBS)
26	112	126	2	Blank (feces)		78	126	2	Blank (PBS)
	102	127	2	Blank (feces)		137	127	2	Blank (PBS)
	140	128	2	Blank (feces)	58	80	128	2	Blank (PBS)
	103	129	2	Blank (feces)		98	129	2	Blank (PBS)
	100	130	2	Blank (feces)		149	130	2	Blank (PBS)
27	97	131	2	Blank (feces)		86	131	2	Blank (PBS)
	75	132	2	Blank (feces)		18	132	2	Blank (PBS)
	13	133	2	Blank (feces)	59	140	133	2	Blank (PBS)
	108	134	2	Blank (feces)		33	134	2	Blank (PBS)
	116	135	2	Low		105	135	2	Low
28	49	136	2	Blank (feces)		4	136	2	Blank (PBS)
	42	137	2	Blank (feces)		120	137	2	Blank (PBS)
	104	138	2	Blank (feces)	60	108	138	2	Blank (PBS)
	84	139	2	Blank (feces)		24	139	2	Blank (PBS)
	48	140	2	High		36	140	2	Low
29	74	141	2	Blank (feces)		79	141	2	Blank (PBS)
	4	142	2	Blank (feces)		135	142	2	Blank (PBS)
	89	143	2	Blank (feces)	61	38	143	2	Blank (PBS)
	85	144	2	Blank (feces)		61	144	2	Blank (PBS)
	80	145	2	High		107	145	2	High
30	106	146	2	Blank (feces)		9	146	2	Blank (PBS)
	64	147	2	Blank (feces)		13	147	2	Blank (PBS)
	53	148	2	Blank (feces)	62	22	148	2	Blank (PBS)
	23	149	2	Blank (feces)		99	149	2	Blank (PBS)
	139	150	2	Low		154	150	2	High
31	121	151	2	Blank (feces)		111	151	2	Blank (PBS)
	133	152	2	Blank (feces)		23	152	2	Blank (PBS)
	3	153	2	high spike level	63	130	153	2	Blank (PBS)
	60	154	2	Blank (feces)		102	154	2	Blank (PBS)
	71	155	2	High		26	155	2	High
32	35	156	2	Blank (feces)		63	156	2	Blank (PBS)

	115	157	2	Blank (feces)		41	157	2	Blank (PBS)
	154	158	2	Blank (feces)	64	150	158	2	Blank (PBS)
	36	159	2	Blank (feces)		127	159	2	Blank (PBS)
	150	160	2	High		73	160	2	Low
N/A	161	161	Archive	Blank	N/A	161	161	Archive	Blank
N/A	162	162	Archive	Blank	N/A	162	162	Archive	Blank
N/A	163	163	Archive	Low	N/A	163	163	Archive	Low
N/A	164	164	Archive	Low	N/A	164	164	Archive	Low
N/A	165	165	Archive	Low	N/A	165	165	Archive	Low
N/A	166	166	Archive	Mid	N/A	166	166	Archive	Mid
N/A	167	167	Archive	High	N/A	167	167	Archive	High
N/A	168	168	Archive	High	N/A	168	168	Archive	High
N/A	169	169	Archive	Blank	N/A	169	169	Archive	Blank
N/A	170	170	Archive	Blank	N/A	170	170	Archive	Blank
N/A	171	171	Archive	Low	N/A	171	171	Archive	Low
N/A	172	172	Archive	Low	N/A	172	172	Archive	Low
N/A	173	173	Archive	Low	N/A	173	173	Archive	Low
N/A	174	174	Archive	Mid	N/A	174	174	Archive	Mid
N/A	175	175	Archive	High	N/A	175	175	Archive	High
N/A	176	176	Archive	High	N/A	176	176	Archive	High

Red text= test samples, High= 1,332 copies/reaction, Low=120 copies/reaction, Blank= no virus/spiked with PBS, pink= Feces, green= PBS, blue= omicron, yellow= WA-1
